# A Little Boat Flopping About on the Ocean: The Lived Experience of Transitioning Early Career Nurses

**DOI:** 10.1002/nop2.70202

**Published:** 2025-03-26

**Authors:** Liz Ryan, Leah East, Andy Davies, Blake Peck, Daniel Terry

**Affiliations:** ^1^ School of Nursing and Midwifery University of Southern Queensland Toowoomba Queensland Australia; ^2^ Centre for Health Research University of Southern Queensland Toowoomba Queensland Australia; ^3^ Institute of Health and Wellbeing Federation University Australia Ballarat Victoria Australia

**Keywords:** early career nurse, imposter syndrome, junior, nurse, support, turnover

## Abstract

**Aims:**

To explore the lived experiences of transitioning from a student to a nurse while navigating the workplace in their first two years within the profession.

**Design:**

A qualitative exploratory study employing Gadamer's hermeneutic phenomenology to inform the interpretive approach, thereby reinforcing the philosophical foundations of the research.

**Methods:**

Twenty‐six early career nurses who participated in an initial study as students were followed up and interviewed 18–24 months after graduating between 2020 and 2023. Semi‐structured interviews were conducted to collect data and were thematically analysed. COREQ guidelines were followed.

**Results:**

Three themes emerged encompassing: Navigating the New World, where early career nurses felt like imposters and ill‐prepared to practise; The real world, where early career nurses are pushed to their limits; and A support network, where early career nurses relied on each other and other key members of the professional team to make it through the transitional period of being a new nurse.

**Conclusion:**

An examination of early career nurses' experiences centred around support, where differences between anticipated and actual support fostered imposter syndrome and feelings of being overwhelmed. While further research to explore the deeper dynamics of the relationships between peers is required, there also remains further research to understand the mechanisms that inform the flow of recently registered nurses out of the workplace or profession altogether. There are opportunities to better link early career nursing peers and capitalise on the empathic nature of these relationships as one solution to workplace and professional turnover.

**Patient or Public Contribution:**

No Patient or Public Contribution.

## 1 Introduction

The transition and development of Early Career Nurses (ECNs) is crucial in the nursing landscape and remains a global concern. Approximately 25% of all nurses completing their undergraduate programmes choose to leave the profession within the first 5 years after graduation, which is a critical issue given the perilous shortage of nurses globally (Tamata and Mohammadnezhad 2023). The departure of nurses early in their careers poses a serious threat, impacting the ability to provide safe and evidence‐based healthcare. Although leaving a health workplace poses a challenge at the local level and can be costly for a health service, it is an insidious precursor to nurses exiting the profession altogether. If a nurse leaves the profession, the indirect loss of knowledge, skills and workforce productivity is exponential, perpetuating further professional and workforce instability (de Vries et al. 2023).

Currently, workforce turnover at the organisational and professional level remains challenging, and research regarding the turnover and retention of ECNs, accounting for a third of all nurses leaving the profession, remains abundant. Despite research conducted since the early 1970s, factors associated with the retention of ECNs have been examined, culminating in a collective position that once the honeymoon phase of their career ends, ECNs become anxious, angry, and confused as they come to terms with the realities of the work environment (Graf et al. 2021; Richard and Kim 2023). This is where the shock and the inconsistencies of the ‘new normal’ observed within the workplace are attempted to be negotiated, and it is a time period when medical errors, multiple job changes, or an abandoning of the profession is at its highest (Graf et al. 2021). Further, the COVID‐19 pandemic introduced additional stressors for ECNs, such as increased workload, emotional stress, and changes in work conditions. These pandemic‐related stressors included dealing with high patient volumes, inadequate resources and the psychological toll of working in high‐risk environments (Lee et al. 2021).

Key drivers of retention and turnover among nurses have been shown to include several factors including work conditions, which include work demands, schedules and the level of control in the workplace (Tamata and Mohammadnezhad 2023). This is followed by the work environment itself, encompassing key factors such as leadership, culture, recognition, reward, decision‐making and social supports (Graf et al. 2021). Lastly, there are also individual drivers impacting retention and turnover, which include factors such as demographic variables and motivation (Moloney et al. 2018). Moloney et al. (2018), in their seminal work, provide additional insights by highlighting that job demands that lead to burnout are a major contributor to nurses wanting to leave an organisation or the profession. Burnout encompasses workload and emotional aspects of work, including incivility, along with experiencing work–life balance conflict.

A mechanism to counter burnout among nursing staff is the provision of resources that impact engagement within the workplace, which leads to a lower likelihood of the intention to leave an organisation or the profession. These are described as Job Resources and encompass collegial, supervisor, and organisational support, autonomy and professional development (Moloney et al. 2018). Conversely, Personal Resources, such as Psychological Capital (Hope, Self‐Efficacy, Resilience and Optimism) and individual organisation fit, whereby the values of the nurse and organisation match, lead to higher job satisfaction and greater retention (de Vries et al. 2023).

To improve organisational and professional retention among ECNs, multiple and targeted interventions in the early years of the profession are used to enhance confidence through mentors and preceptorships, residency programmes or transition programmes (See et al. 2023). Although transitional programmes have had a positive impact, there remain uncertainties regarding the efficacy and breadth of impact these approaches have had on retention over the past three decades (Halter et al. 2017). On the other hand, interventions that focus on developing transformative nurse leaders to impact group cohesion, investing in the workforce and developing and continuing staff engagement have had positive impacts on ECN retention (Brunetto et al. 2022).

Recognising that multiple interventions have been shown to have the greatest impact, particularly those that emphasise increased autonomy, fewer constraints in direct patient care, and improved work environment (de Vries et al. 2023). However, understanding which interventions are effective in reducing turnover is vital if we genuinely seek to redress the rate of nurse attrition. Despite the large body of evidence, current studies and interventions are narrow in scope (Kenny et al. 2021). Nevertheless, there have been some insights and advances regarding retention being impacted by key factors such as effective workplace relationships, perceived support, attachment or passion for work, autonomy and work–family conflict. In addition, there have been some demonstrable inroads associated with training Nurse Unit Managers to build Psychological Capital among nurses to influence workplace retention (Brunetto et al. 2022).

Extensive resources, both human and financial, have been directed toward investigating how healthcare organisations and specific interventions can positively impact nurse turnover rates (Marufu et al. 2021). However, despite research to date, the anticipated shortage of nurses worldwide implies a limitation in current policies and suggests an incomplete grasp of the underlying challenges (Marufu et al. 2021). This suggests that there is more to understand concerning the challenges that ECNs encounter to better ‘plug the leaking hole’ plaguing the profession.

New research is urgently required to improve our understanding of what capabilities and resources health services possess to recruit and retain nurses; the strategies or interventions that are in place to support nurse retention and those which have the greatest efficacy, if any, to mitigate nurse retention and turnover (Kenny et al. 2021). Further, critical research is also required to test novel practice interventions at the individual staff level to examine the actual or intended turnover rates among ECNs.

However, before an examination of implemented strategies and interventions to support ECN retention is undertaken, there remains a need to explore what the pressing issues are, what it is really like and how ECNs themselves, including those within the first 2 years, navigate the workplace and profession. Current literature has explored ECNs with a greater focus on those with less than 12 months’ experience (Graf et al. 2021; See et al. 2023). While there are several studies that have explored the transition of ECNs, including and beyond their first year (Richard and Kim 2023), the current data remain limited in relation to the lived experience and finding of support networks among ECNs. This underscores the necessity for a deeper exploration of the intricate facets of ECNs longitudinally, encompassing an investigation into the lived experiences of new nurses throughout their professional careers, including a more nuanced and deeper understanding of what the contributing factors are that may impact their desires to stay or leave their workplace or the profession altogether.

## 2 The Study

### 2.1 Aims

Within the context of the current literature, the aim of the study was to explore the lived experiences of transitioning from a student to a nurse, while navigating the workplace in their first 2 years in the profession.

### 2.2 Design

A qualitative exploratory study designed and informed by the theoretical tenets of hermeneutic phenomenology as outlined by Gadamer (2013) was used to explore the experiences of ECNs in Australia and the impact of their transition, learning, and future career choices in nursing. At its core, Gadamer's ideas and assumptions suggest that we each come to our own understanding of the world and that the condition of this understanding is language. In addition, this language is not different from the language we share with each other, and as such, we are able to understand the experiences of others through deeper dialogical engagement. Therefore, the hermeneutic phenomenological approach enables the description and interpretation regarding the fundamental structures of the lived experience of participants (Gadamer 2013). To achieve this, ECNs were invited to participate in a longitudinal follow‐up study initially conducted by Terry and Peck (2020). Reporting methods adhered to the COREQ guidelines.

### 2.3 Participants

Twenty‐six early career nurses who participated in an initial study as students (Terry and Peck 2020) were followed up and interviewed 18–24 months after graduating between 2020 and 2023 (Table 1). Participants were recruited from a diverse range of healthcare environments, including metropolitan, rural, and remote settings, to ensure a representative sample of ECNs. Recruitment was conducted through email invitations sent to former third‐year nursing students who had participated in the initial study and had indicated their willingness to be contacted for follow‐up interviews.

**TABLE 1:** Interview of ECRs over four‐year period.

Graduated	2018	2019	2020	2021	
Year of interview	2020	2021	2022	2023	Total
Early career nurse	9	6	8	3	26

To mitigate selection bias, efforts were made to include participants from various demographic backgrounds, including different age groups, genders and healthcare settings. The high response rate (93%) suggests a strong willingness among participants to share their experiences, which enhances the credibility of the findings. However, potential biases may still exist, such as self‐selection bias, where those who had particularly positive or negative experiences may have been more likely to participate. To address this, the study ensured confidentiality, encouraging honest and open responses. Additionally, the use of semi‐structured interviews allowed for a comprehensive exploration of participants' experiences, reducing the risk of missing critical insights.

### 2.4 Data Collection

Data were collected mid‐year between 2020 and 2023. The two‐year follow‐up interviews were based on the year the participant had graduated. For example, 2018 university graduates were interviewed in 2020 and so on, as outlined in Table 1. Interviews were conducted via telephone in 2020 (*n* = 9), while the remaining interviews were conducted via videoconferencing technology from 2021 onwards (*n* = 17) which related to greater access to technological advances over time. The semi‐structured interview frame, which had been piloted, included several standardised questions, such as employment history since graduating, the experience of nursing, challenges of the profession and positives associated with nursing. Further, participants were also given the opportunity for an open dialogue with the interviewer about their lived experiences as ECNs and what it meant to transition from a student to a nurse and their experiences of navigating the workplace, the culture and managing their personal lives. Data saturation was determined when new themes were no longer highlighted and new responses were not generating additional understanding. It must be noted that the 2020 data collection occurred within weeks of the international public health emergency, Coronavirus Disease (COVID‐19). Data were collected by one researcher (XX), who specialises in health workforce, and lasted between 20 and 60 min. As guided by the study protocol, which outlined the steps undertaken from the start of the longitudinal study to the development and reporting of findings, fieldnotes were taken throughout and after the interviews were conducted. Further, peer debriefing occurred throughout each data collection period between researchers (XX) and (XX).

### 2.5 Data Analysis

Data were initially transcribed into Microsoft Word in 2020; however, with the commencement of videoconferencing interviews, transcription was provided through technology thereafter. Data were cleaned, and member checking was invited for accuracy. Transcripts were provided; however, no participants provided additional commentary, correction or insights regarding their interview. Data were labelled according to the year the participant was interviewed and their interview order (e.g., Participant [P]2, 2020; P16, 2022; P21, 2023). Prior to data being pooled, the data set from each year was examined to compare the data collected via telephone in 2020 and all later data collected via videoconferencing to ensure data similarity.

Thematic analysis was used to deduce the themes within the data corpus (Braun and Clarke 2022). The researchers followed the six phases of Thematic Analysis which commenced with immersing themselves in the data by reading and re‐reading the interview transcripts and generating initial codes. Meanings were then assigned to each data set and each of the significant quotes from the interviews were grouped into themes independently by three researchers (XX, XX and XX) until consensus was achieved. This step involved sorting the different codes into themes and collating all relevant coded data extracts within the identified themes. The themes were further refined through collective discussion and agreement within the research team. The themes were named in a way that defined their essence using participants’ excerpts to enhance confirmability. This process ensured a systematic approach was undertaken to the data analysis and was chosen as it does not consider previous research outcomes and minimises researcher bias, therefore improving research trustworthiness (Braun and Clarke 2022) (Table 2).

**TABLE 2:** Example of codes, themes and illustrative quotes.

Theme	Codes	Relevant quotes
Navigating the new world	Feeling inadequate and unprepared when entering the workforce.	‘I kind of felt like I was bullshitting my way through it for the first three or 4 months’ (P16, 2020).
	Experiencing self‐doubt and questioned their abilities.	‘It was harder to deal with my own head than dealing with the patient… the feeling of inadequacy, being a new nurse, dealing with a patient that's deteriorating, and feeling like an imposter… I cried’ (P6, 2022).
The real world	Overwhelmed by the workload and felt constant pressure.	‘You could never catch up, never get on top of your workload… drowning’ (P14, 2022).
	ECNs felt they did not receive adequate support from peers and senior staff.	‘You got told that there would be support, but there was not support due to staff shortages’ (P14, 2022).
	Experiencing bullying and negative behaviours from other staff.	‘The bullying was awful in the medical ward, and you felt set up for failure. Felt belittled, roared at. Lots of crying… Very “cliquey”… felt targeted, and not supported…. Lost confidence in self’ (P21, 2021).
A support network	Relying on fellow ECNs for social and emotional support.	‘We [ECNs] used to just catch up and check on one another and see how you're going and, it was particularly in that first 6 months of feeling like a fish out of water… You talk to someone else they're feeling exactly the same way you are; it made a big difference’ (P8, 2020).
	Support from mentors and educators was crucial for ECNs' transition.	‘The support of the team. I could nurse anything I think, any patient with that team behind me, and if my NUM moves anywhere in the world, I think I'd follow her, I really would… I think most of us would do the same. She cares for her team deeply’ (P3, 2020)’
	Relying on family and friends for support during challenging times.	‘It's great just to hear how everyone's experiences are, where they are hoping to end up, what their plans are, hearing any interesting case, anything that's a bit out of the ordinary that you might not necessarily come across’ (P10, 2020).

Thematic saturation was determined when no new themes or sub‐themes emerged from the data, indicating that additional data collection would not provide further insights (Carpendale et al. 2017; Saunders et al. 2018). This was achieved after a thorough analysis of the interview transcripts, where recurring patterns and themes were consistently identified across multiple participants. If unexpected themes were identified during the analysis, they were carefully examined and incorporated into the findings if they provided significant insights into the research questions. If any unexpected themes were deemed not directly relevant, they were documented and excluded from the final analysis, with justification provided by the research team for excluding unexpected themes. This approach strengthened the credibility of the results of the study by ensuring a comprehensive and transparent analysis process (Braun and Clarke 2022; Carpendale et al. 2017; Saunders et al. 2018).

### 2.6 Ethical Considerations

Ethical approval for the study was procured from Federation University Human Research Ethics Committee (Approval #18–017). All aspects of the research adhered to the ethical principles for medical research on human beings, as set out in the Declaration of Helsinki, and all methods were performed in accordance with the relevant guidelines and regulations. Each participant provided informed consent prior to commencing data collection.

## 3 Findings

Participants were a heterogeneous group with the demographics indicating the majority (85%) were female, aged between 20 and 39 (66%), and were currently working in a hospital setting (76%). Of the participants, 20 participated in a graduate programme as part of their first year after university studies; six did not complete their graduate programme. Graduate programmes, designed to support newly registered nurses to transition into practice, varied between two or three rotations relative to the health service graduate programme being provided by individual hospitals or health services. The rotations were between four and 6 months and based on individual ECN preferences; however, being matched with preferences was not always possible. In most cases, those who did not complete their graduate programmes were offered permanent positions where they were currently working. Others were offered positions in areas of nursing they preferred or had taken up opportunities in nursing elsewhere due to personal circumstances. The areas where participants were currently working were diverse, with 38% (*n* = 10) of participants working in rural or remote areas, while 8% (*n* = 2) were not currently working due to illness or leaving nursing altogether, as outlined in Table 3.

**TABLE 3:** Participant characteristics.

Demographic information	2018	
	*n* = 26	(%)
Sex		
Female	22	85%
Male	4	15%
Age group		
20–29	9	35%
30–39	8	31%
40–49	6	23%
50–59	3	12%
Graduate year		
Completed	17	65%
Incomplete	6	23%
Did not participate in graduate programme	3	12%
Where currently working		
Hospital	20	76%
Aged Care	2	8%
Community	2	8%
Not currently working as a nurse	2	8%
Where currently working now—geography		
Metropolitan/Urban	14	54%
Rural/Remote	10	38%
No applicable	2	8%
Hours currently working		
0.9–1.0	3	12%
0.7–0.8	16	62%
0.5–0.6	2	8%
Casual	3	12%
Not applicable	2	8%
Area of nursing in current role		
Emergency	1	4%
Mental health	3	13%
Medical/Surgical ward	6	25%
Maternity/Neonatal Intensive care	3	13%
Oncology/Palliative care	3	13%
Aged care	2	8%
Community health	1	4%
Theatre/Day procedure	1	4%
Dialysis	1	4%
Pool	3	13%
Enrolled nurse background	3	12%

**FIGURE 1:**
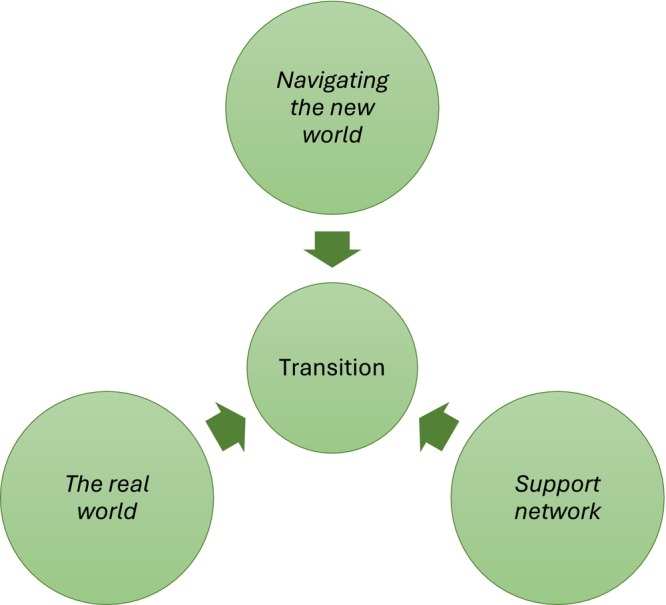
Key themes identified associated with the transition of early career nurses.

Three themes emerged from the participants narratives. The first theme, *Navigating the New World*, encapsulates how the ECNs felt like imposters and ill‐prepared to practise as newly graduated nurses. The second theme, *The Real World*, reveals how the participants were pushed to their limits during the first 2 years of their nursing career, highlighting the stress and pressure they experienced. The third theme, *A Support Network*, reveals how the participants relied on other ECNs and key members of the professional team to make it through the transitional period of being a new nurse (Figure 1). Each theme is discussed individually.

### 3.1 Navigating the New World

Although being 18–24 months since graduating, the ECNs in this study commonly reflected on and described themselves as an *imposter* and feeling *inadequate* when they entered the workforce as new graduate nurses. These feelings, raw for many who still felt like new nurses, commonly stemmed from a *lack of confidence*, which translated to a sense of inability to provide patient care as a registered nurse. Common threads through the ECNs narrative, when reflecting on being newly graduated nurses, were feeling it was ‘*horrible, dangerous’* (P17, 2022), ‘*a baptism of fire’* (P6, 2022), a ‘*train wreck, bonkers, scary*’ (P22, 2021) or that they felt like ‘*a fish out of water’* (P8, 2020) or ‘*a little boat flopping about in the ocean’* (P16, 2020). Narrative reflections were characterised by the participants sense of being ill‐prepared and overwhelmed, which highlights the difficulty these ECNs experienced as they transitioned from a student nurse to a registered nurse. The following comment was common through many participants' narratives when reflecting on first entering the profession and even now, 2 years later:

‘*horrible… I wasn't fully prepared for what I was getting myself in for at all. The learning curve was really steep… I still felt like a student…’* (P18, 2020).

This was further collaborated by another participant who found the experience of being an ECN initially overwhelming, when stating ‘*I hadn't had any real acute experience of people who are sick…I thought I was going to kill somebody every day. I thought they were going to die’* (P16, 2020).

Participants expressed that they were constantly questioning themselves which some found mentally challenging and exacerbated by the feelings of being an imposter. For example, on reflection, one participant stated *‘I kind of felt like I was bullshitting my way through it for the first three or four months*’ (P16, 2020) Likewise, another participant shared:

‘It was harder to deal with my own head than dealing with the patient… the feeling of inadequacy, being a new nurse, dealing with a patient that's deteriorating, and feeling like an imposter… I cried’ (P6, 2022).

Further, participants had to negotiate the transition from being a student to an ECN with responsibility, and part of this was the realisation of not having the safety of a fellow registered nurse overlooking the care being provided, which exacerbated the feelings of being an imposter:

‘You feel like an imposter, I guess, for the first few weeks you're doing meds without someone standing beside you. Your like, “oh no, I can do that now.” I'm not going to get in trouble for that. I'm not going to have someone tap me on the shoulder and be told to go home’ (P6, 2022).

Likewise, feelings of being an imposter and the difficulty that can occur during transition is the nature and tension of being a dependant student through to being an expected independent practitioner. ECNs were still feeling this tension 2 years after graduating:

‘It is that thinking – “I'm all on my own here. I've got to make decisions without somebody looking over my shoulder the whole time – now, why did you do that”… obviously there is support there but you've got it in the back of your head thinking – “am I doing the right thing here”?’ (P20, 2020).

Reflecting on their undergraduate education, participants postulated feeling ill‐prepared for the workplace and nursing profession, which contributed to their self‐doubt. One participant, when talking about what they knew and did not know stated:

‘*I had the knowledge and preparation not to do anything stupid, but there were times where I feel I didn't have the knowledge to adequately answer questions that were presented to me from my patients’* (P18, 2020).

It was suggested that ECNs ‘*don't know what they don't know’* (P4, 2020) and in these situations you are learning through experiences. One participant expressed:

‘*It's a really big skill to have, how to really care for someone while they're dying and after they've died… having difficult conversations with family and supporting them through it. You have to learn as you go’* (P4, 2020).

Working with families and dealing with emotions was highlighted as areas where the participants felt specifically ill‐prepared. For example, a participant provided the following reflective insights:

‘W*e were never taught how to deal with aggressive family members – people that are in your face* [while] *you've got 20* [other] *things to do and they're asking you 50,000 questions and why something wasn't done. You're not taught…. that you're allowed to walk away. So, you have to learn that on your own’* (P18, 2020).

However, participants at times found that their age and life experiences were beneficial in coping emotionally with the transition, where a participant aged in her 50s stated:

‘*If I wasn't the age I am, after that first day I reckon I would have just walked away and not gone back. If I was a young girl, fresh out of uni, just moved out of home from mum or dad or something, it would have just crushed my soul and I would have just gone’* (P21, 2021).

Highlighting the need to be prepared for the emotional labour of nursing, one participant stated: *‘*[We] *should be taught how to take care of yourself emotionally within those first few months’* (P18, 2020). The same participant explained that it is the emotional work associated with the profession that leads to individuals leaving the profession:

‘*I know a lot of people that were in my grad*[uate] *year that left because they couldn't handle it…. the dropout rate is high because we're just not given the appropriate tools, such as dealing with challenging behaviours, and emotional intelligence*’ (P18, 2020).

Similar to attributing undergraduate education to feeling ill‐prepared, the participants also expressed that the orientation and support in the workplace afforded by graduate programmes did not meet their learning needs. For example, the following comment was not uncommon throughout participant narratives:

‘*In orientation, I was sitting there going ‘what I am I doing?’ The first week or two you are trying to take blood pressures and things like that, when you can't hear* [it] *and then you start to second guess yourself’* (P11, 2020).

In addition to the challenges with settling in and questioning themselves, another participant reflectively added:

‘*… there is a lot to take in in a short period of time to be able to be independent…. You are classed as independent after three shifts, but you're still asking questions every five minutes*’ (P11, 2020).

Upon reflecting on an experience associated with an acute care setting, one participant highlighted the generalist nature of undergraduate education combined with limited supernumerary training can equate to being ill‐prepared:

‘*We were given two supernumerary days in the special care unit and going from no experience as in undergraduate studies to having to nurse babies was really difficult…I knew how to change a nappy, hold, feed and do basics…a lot of* [the] *students that I started with had nothing to go on, so they were learning right from the start*’ (P2, 2020).

Participants outlined there were so many *little things* that they did not know and needed to understand. As outlined in the following excerpt, participants expressed a gap in their experiences and knowledge which they perceived could have been provided through support and greater orientation:

‘Someone would come in on night shift and say – have you done all this for your patient and… I didn't realise I had to? No one ever told me. When I did my orientation, I was just like a lapdog and the nurses just used me to do stuff they didn't want to do, but never really taught me anything’ (P21, 2021).

### 3.2 The Real World

The second theme that emerged from the analysis of the participants’ experiences encompassed *The Real World*, which embodies the way in which ECNs had built a perception of what being a registered nurse would be like and that this was not in fact the reality that they confronted. Instead, they remain faced with pressure, lack of support, and bullying behaviours. ECNs, when reflecting on their time since graduating until now, were both drawing on past and present experiences, suggesting that the transition from student to nurse was still entrain.

Overall, the ECNs indicated that they were overwhelmed by the workload, limited support, and limited staff, where they felt they were ‘*beaten down…* [and]*… had to go with the flow to survive’* (P2, 2020). Overwhelmingly, many had expressed that as they transitioned through their graduate programmes, they felt that they did not have the ability to make any changes or improvements. A participant who worked in a medical ward found it to be so busy and stressful, expressed that it was ‘*like you could never catch up, never get on top of your workload… drowning’* (p14, 2022). Participants indicated that they use break times to try and catch up with the workload, and that ‘*the stress of feeling you have to get everything done…* [you] *do your best… it was just that overwhelming stress’* (P18, 2020). Another participant added:

‘*If you've got IV after IV and you're… aware of those medications that will have* [to be] *administered… it's tricky… You know everyone else might be quite busy… so it's* “*oh, I'm running behind… help”*’ (P26, 2023).

Participants, when reflecting on their experiences as an ECN found that, you ‘*got told that there would be support, but there was not support due to staff shortages’* (P14, 2022). The stress felt and seen among ECNs and the nursing profession was evident for a participant who shared: *‘Two out of five days* [a week] *people left crying’* (P17, 2022).

Participants described being continually worn out and under constant pressure, and this led to feelings of wanting to leave the workforce and profession. For example, comments such as ‘*You could easily walk out and not come back’* (P2, 2020) and knowing others who had left ‘*due to a horrible grad experience’* (P17, 2022) were replete throughout the participants transcripts. This ECN's experience in the acute care setting created anxiety, and took an emotional toll, leading them to leave nursing for several months, and later returning to aged care, vowing not to work in that acute care area again (P17, 2022):

‘*I was so stressed out and so… I'm worried that something's going to go wrong because you were just put under such pressure…. Nothing would go in* [mentally]*… I decided I would resign from the graduate year before I killed someone… I had the benefit of walking away and straight back into my old job. Not everyone has that. So, a lot of them were crying every day hoping to get through this* [year] *anyway* [they could]’.

Other participants spoke of the struggle with clinical practice, feeling constantly rushed and facing difficulties with the complexity of the patients that they were allocated (P13, 2022):

‘I think when you're studying, you don't have the same time constraints as you do when in the practical sense, everything is really rushed in the real world’.

The lack of support perceived by the participants, combined with the feelings of stress and pressure equated to them feeling they were not able to fully fulfil their role as a registered nurse. For example, a participant expressed: ‘*You can't learn because you are so stressed—under so much pressure’* (P17, 2022). Staff ratios were also raised as hindering participants’ ability to fulfil their roles: ‘*you can't give people the care that they need. Everyone is screaming for help, but there was no one to help’* (P14, 2022). Likewise, another participant stated *‘Other people can't help you, or you them. There is so much pressure’* (P17, 2022). To cope and get through these times, P14 shared they just had to get through the year: ‘[You] *keep telling yourself that it is only a year, that it is all a learning experience*’ (P14, 2022).

One participant found that they did not get the support from educators as expected:

‘*They were nurses. They didn't have time to be educators because we were so short staffed. So, I was like – ok, I need focus and help here. Like you had to become very proactive in what you needed help within that graduate year…*. [and] *how you dealt with it. There were a few that weren't proactive that really did struggle and dropped out*’ (P6, 2022).

Exacerbating feelings of being pushed to the brink, participants shared experiences of feeling bullied and belittled by other staff. P4 described a bullying culture within their graduate year, and that this promoted an unhealthy work/life balance, noting the participant also left the programme:

‘My first rotation there was a big bullying culture on board…. That can be a lot having to go into work every day knowing that you have to be put in situations with people like that…. I enjoyed the specialty, but the culture was less than ideal. It was like – “We don't take breaks because we prioritise patient care. Oh no, we don't call in sick because we prioritise patient care.” They just had a really unhealthy work/life balance culture’ (P4, 2020).

Other participants were aware of the historic rhetoric associated with nursing and stated a negative culture was caused by individuals such as ‘those with superiority complexes’ (P11, 2020), and ‘*nurses who have been in the job too long, and not prepared to change*, [and] *who were critical of each other… nurses eat their own, but that*'*s just history*’ (P16, 2020). P21 (2021) stated:

‘The bullying was awful in the medical ward, and you felt set up for failure. Felt belittled, roared at. Lots of crying*…* Very “cliquey”… felt targeted, and not supported…. Lost confidence in self’.

On reflection, another participant shared that although professional conflict can be cruel it can have the benefit of making you stronger:

‘*Staff can be cruel. You must know everything, or they would ride you. Or* [will not] *talk to you… went home crying often. Teaches you to be stronger though*’ *(*P23, 2021*)*.

Others felt that when they expressed concern, they were not listened too, because of their status as a junior with little experience:

‘People say you're a baby nurse, just sit in the corner…. You really had to push…I'm a grad, but I'm also a nurse, I'm not silly…. I was really concerned about this patient, but nothing was really done about it…they ended up on airway support six hours later’ (P6, 2022).

One participant found they did not get as much support as other ECNs, as they were known to be a previous enrolled nurse. In this situation, it had been presumed that they did not need support and they would be able to manage in their new professional role. This, however, was not the case:

‘*I think we* [previous enrolled nurses] *were left to stand on our own two feet a little, as opposed to those who had no previous experience. The educators go*, ‘*well, they*'*re older nurses. They*'*ve been enrolled nurses for many years. They*'*re probably not needing as much* [help] *as others*.’ *But sometimes you really need that help and support*’ (P12, 2021).

Although some participants expressed that they had experienced bullying, some also were able to advocate for themselves and were not afraid to stand up for themselves, For example, one participant stated: ‘*I*'*m a very forthright person, so I will say –* “*Well, hang on a minute, where*'*s this coming from?*” *You sort of call it out*’ (P12, 2021). Many participants also felt that being of an older age made it easier for them to deal with things, to speak up if they needed to, or not engage in negative behaviours, including bullying: ‘*I have had the benefit of being around a fair while, knowing stuff pretty much works out in the end*’ (P16, 2020) and ‘*I was 50 when I started, so I just toughed it out. Whereas you know that*'*s where we lose some of them because they don*'*t have the strength to face up every day*’ (P21, 2021).

### 3.3 A Support Network

Regardless of the challenges associated with feelings of inadequacy, feeling unprepared and feeling bullied, participants indicated that support from fellow ECNs and team members, when available, made a positive impact on their transition period. Participants found that getting together with fellow ECNs was helpful in ‘surviving’ the initial first year. They were able to support each other socially and emotionally and know that others were feeling the same way or having similar experiences. Participants found it positive to be able to debrief with each other about how they were going. There was a shared understanding of what it was to be a newly graduated registered nurse, which they could not share with others. They could also mentor each other as they grew and learnt in their positions. For example, one participant stated:

‘*We* [ECNs] *used to just catch up and check on one another and see how you*'*re going and, it was particularly in that first six months of feeling like a fish out of water… You talk to someone else they*'*re feeling exactly the same way you are; it made a big difference*’ (P8, 2020).

Another participant explained they were working with a lot of ECNs, and they could debrief and support each other, ‘*just having that person that was in the exact same position as you, in those wards dealing with the same people. You could rely on to debrief and chat*’ (P2). Fellow ECNs were supporting each other and had expressed the importance to stay connected with each other. Specifically, one participant stated:

‘*It*'*s great just to hear how everyone*'*s experiences are, where they are hoping to end up, what their plans are, hearing any interesting case, anything that*'*s a bit out of the ordinary that you might not necessarily come across*’ (P10, 2020).

Other participants used friends and family as supports after having a negative time, as they did not want to share with fellow ECNs as they ‘*didn*'*t want to be seen as a gossip*’ (P21, 2021). It was important to find a support network, whatever that may be, and to be prepared for challenges on a personal, as well as a professional level. It was suggested that ECNs should not ‘*think that at some point you are not going to need a support network*’ (P6, 2022). Later this participant added:

‘*Don*'*t go into it* [nursing] *with rose coloured glasses thinking it*'*s going to be great. Be prepared to be shaken to your core with things that will happen, you will see, and deal with*’ (P6, 2022).

In addition to getting support from other ECNs, family and friends, participants found that having support from fellow staff and educators made a difference in their ability to cope and get through each shift. Many discussed that support was dependent upon who they were working with on any given day. Some expressed fantastic support from educators and Nurse Unit Managers (NUM), as well as fellow floor staff. For example:

‘The support of the team. I could nurse anything I think, any patient with that team behind me, and if my NUM moves anywhere in the world, I think I'd follow her, I really would… I think most of us would do the same. She cares for her team deeply’. (P3, 2020).

Camaraderie in the clinical environment was important and a place to gain support from others. P14 (2022) expressed the impact mentors can have. This participant, when discussing having a mentor in the Post Anaesthesia Care Unit (PACU) where they worked, stated:

‘*She put me in charge, then made me go faster*. “*What are you doing now? Tell me*.” *It was so stressful and thought I was going to have a heart attack. By the time I finished, I*'*d got all these patients safely through PACU. An amazing day and we got to the end. And she*'*s like* “*You survived, and you did an amazing job*.” *Probably one of the best shifts I worked. Pushed to the absolute limits*’ (P14, 2022).

Support also came from the educators, who were able to advocate on behalf of the ECNs (p6). ‘*I had a great educator—she was amazing, like she really fought. Really really fought for us not to do extra shifts, the double shifts. And another—she was really good. She*'*d go*, “If you need anything, you know where my door is.” *She*'*d come help me and things like that*’ (P6, 2022*)*. Fellow staff members, buddies, and nurse unit managers were also very supportive to many ECNs.

One participant found that working in a smaller hospital made access to educators easier, and the support from other staff was positive.

‘We had support. It was so easy to get hold of an education of the grad[uate] coordinator for the programme because it's such a small hospital. You don't have to fight for the same number of educators attention if you need something’ (P20, 2020).

The participant later adding that working in an operating theatre it was ensured that ECNs were always buddied, however, was often dependant on who they were working with:

‘It depended on who you were buddied up with for that shift as to how much freedom, as it were, to do things—to adapt your practice to how you found it worked…the majority of staff were very supportive’ (P20, 2020).

In addition, another participant shared similar experiences when working in mental health. It was indicated that support depended a little on who they were working with at the time, finding that older nurses were less inclined to assist sometimes.

‘It depended on their experience and their age as nurses. When they're a bit older they brush things off because I think maybe they've seen it all. When they're a bit younger they may be held your hand a little bit more and helped you out with things’ (P18, 2020).

Another participant stated that they got to know which staff were helpful and those that were not. They found that some ‘*staff don*'*t trust you when they hear that you haven*'*t got much experience, so they watch you, or take over rather than support and teach*’ (P23, 2021), highlighting the importance of mentorship. Lastly, some participants found the connections they form with patients and their families is something that helps get them through also. One participant indicated:

‘I've had a lot of positive experiences… I would say the positive experiences you have when you get that positive feedback from your patients, when you can see them smile and you know you've made a difference with them’ (P6, 2022).

Overall, there was consensus among most participants that their patients were at the centre of what they did and that there was satisfaction that they were able to make a difference. When receiving positive feedback from patient and families, this was a reminder of why they chose to pursue a career in nursing.

## 4 Discussion

This study sought to explore the lived experiences of transitioning from a student to a nurse while navigating the workplace within their first 2 years in the profession. This study offers a nuanced understanding of ECNs experiences of this period of change and provides rich insights into those factors that matter from the perspective of the nurses themselves. The findings suggest that the experiences of ECNs participants are embodied by way of three main areas, including their feelings of being an imposter, the stark contrast between their expected reality and the actual reality of nursing, and the lack of effective support that was on offer. These insights offer an opportunity for a more focused approach to addressing these issues by education providers as well as healthcare agencies themselves. The ways that these findings can inform practice as well as the way in which these ideas are situated within the literature will be examined here.

Early Career Nurses within the study identified a sense of being unprepared and overwhelmed by the experience of transitioning from student nurse to registered nurse. While this transition represents a documented period of upheaval for newly graduated nurses (Naylor et al. 2021), the participants were able to articulate a more nuanced understanding of their experiences and emphasised a sense of questioning their own capacities. Feeling unprepared may be related to how other ECNs have felt unprepared within the literature (Graf et al. 2021; Richard and Kim 2023). However, given the exceptional circumstances of COVID‐19, these feelings of being unprepared may have been further exacerbated for all within the first 2 years of graduating, just after graduating and even among students who entered the workforce after the pandemic.

However, the transition experience for ECNs can vary widely due to cultural and systemic differences. Cultural backgrounds may have an impact on communication, expectations, and workplace interactions, posing challenges like language barriers and differing social norms (Baharum et al. 2023; Schumann et al. 2024). These factors can impact ECNs' confidence and team integration. Promoting cultural competence through sensitivity training and fostering an inclusive environment remains essential. Systemic factors, such as healthcare policies, organisational culture and resources, also influence ECNs' transition (Leyva‐Moral et al. 2023). Under‐resourced settings can lead to higher workloads and limited mentorship. Discrimination based on race, gender or socioeconomic status can further complicate the process (Jolley and Peck 2022).

Further, the feelings of self‐doubt among ECNs are embodied by the idea of imposter phenomena (IP) or Impostorism (Barrow 2019), a phenomenon that has experienced a growing body of evidence to support its relevance to the nursing profession. Work by Barrow (2019) and Edwards‐Maddox (2023) for example, identified that imposterism might be linked to accelerated burnout and turnover, particularly among the ECN group of nurses. Interestingly, both studies highlight a complete dearth of research that focuses on this particularly vulnerable population of transitioning nurses. The present study, therefore, would support the idea that IP is experienced by ECNs interviewed and represents one element in the conundrum of nurse attrition that warrants a more focused examination.

The participants in the present study identified that staff shortages, as a precursor to higher workloads, were mediated in the context of perceived levels of support. It is important to recognise that many participants in this study have endured the COVID‐19 pandemic as ECNs, where there was a surge in nurses wishing to leave the profession due to a number of key factors that encompass pandemic stress and higher workloads (Lee et al. 2021). ECNs in the present study described a sense of not feeling well supported by their peers during their period of transition. While this is often reported in existing literature (Salisu et al. 2019), the transition of the participating ECNs in the current study coincided with unprecedented levels of staff being furloughed in light of the pandemic and associated need for reprioritisation of resources (See et al. 2023). The sequelae of this is higher workloads for under‐prepared staff and decreased or redirected senior nursing staff who are therefore unavailable to provide much‐needed support.

ECNs described the lack of peer and more senior nursing support as creating the conditions where bullying can thrive. Workplace incivility in nursing has an unfortunately established history (Cortina et al. 2022); however, a body of research now highlights the fundamental role that daily social support from both managers and colleagues can have as a resource to reduce the likelihood of incivility spirals (Liu et al. 2021). Interestingly, the mature age ECNs in the present study suggested that their age and life experience meant they were sufficiently prepared to advocate for themselves and to be forthright in their requests for support. In so doing, they avoided negative behaviours within the workplace such as bullying—a finding that has been identified elsewhere (Carmona‐Cobo and Lopez‐Zafra 2022), but warrants targeted investigation and intervention.

Consistent with the work of others, ECNs in the current study identified the need for support from others in order to provide safe and effective patient care (Richard and Kim 2023), and there is a mismatch between their expectations and the reality of work and the workplace (Bakker et al. 2019). An umbrella review by Kaldal et al. (2023) concluded that it remains unclear as to the support resource that is of most utility to ECNs. Despite this, participants in our study identified that it was time spent with their peers that was of most value. Peers provide lived experience, empathy, and know that others are feeling the same way.

This capacity of peers to support one another was considered a tremendous strength, and given it has been demonstrated that peer‐to‐peer learning and support between junior and senior nursing students is essential (Terry et al. 2022), we can postulate that this practice would occur among ECNs. Given the value placed on support as a possible mechanism of nurse career retention and longevity, further work is needed to find formalised mechanisms for peer‐to‐peer support and to better understand opportunities for building a community of practice as a normal part of nursing work. Also, research may be needed to determine if studies conducted during the pandemic are generalisable to non‐pandemic contexts. It is vital to explore whether the interventions and support mechanisms developed during the pandemic are effective in non‐pandemic settings.

The experiences of ECNs in this study can be understood through the lens of several established theoretical frameworks on professional transition and workplace adaptation. One such framework is Bridges' Transition Model (2019), which delineates the process of transition into three stages: (1) Ending, Losing, and Letting Go; (2) the Neutral Zone; and (3) the New Beginning (Bridges and Bridges 2019; Musamali 2018). The feelings of being an imposter and the stark contrast between expectations and reality experienced by ECNs align with the first stage, where individuals must let go of their old identity as students and face the uncertainties of their new roles as registered nurses. The Neutral Zone, characterised by confusion and distress, is evident in the participants' narratives of feeling overwhelmed and unsupported. Finally, the New Beginning stage, where individuals start to embrace their new roles, can be linked to the support networks that ECNs relied on to navigate their transition (Bridges and Bridges 2019).

Similarly, Schlossberg (1981) provides another relevant framework, emphasising the importance of support systems, coping strategies, and the individual's perception of the transition (Schlossberg 1981). The findings of this study highlight the critical role of support networks, both formal (e.g., mentors, educators) and informal (e.g., peers, family), in helping ECNs cope with the challenges of their new roles and align with Schlossberg's assertion that support systems are crucial for successful transitions. Additionally, the feelings of inadequacy and imposter syndrome reported by ECNs can be understood through the lens of Bandura's Self‐Efficacy Theory (1977), which posits that individuals' beliefs in their capabilities influence their ability to perform tasks and handle challenges. The lack of confidence and self‐doubt experienced by ECNs may be indicative of low self‐efficacy, which can be addressed through targeted interventions aimed at building confidence and competence (Bandura 1977).

In addition to these frameworks, the Transition to Practice (TTP) Model offers further insights into the experiences of ECNs. The TTP model focuses on the structured support and development of new nurses through formal programmes that include mentorship, clinical supervision and ongoing education (Spector et al. 2015). This study highlights the importance of support networks, and the challenges faced by ECNs align with the principles of the TTP model. The lack of adequate support and the feelings of being overwhelmed reported by participants suggest that more structured and comprehensive TTP programmes could help mitigate these issues. In addition, healthcare organisations may also consider implementing policies promoting equity and inclusion, ensuring adequate staffing and providing targeted support for ECNs with cultural and systemic differences (Müller et al. 2024). Inclusive practices, such as cultural competence training, mentorship programmes and support networks, can enhance ECNs' transition to professional practice, contributing to a more diverse and equitable healthcare workforce that may be beneficial in the transition period (Craft‐Blacksheare 2018).

In addition to the TPP model, Duchscher's Transition Shock Model (2009) describes the initial period of transition from student to professional nurse as a time of significant stress and adjustment. This model identifies stages of transition shock, including doing, being and knowing, which align with the experiences of ECNs in this study (Duchscher 2009). The feelings of inadequacy and imposter syndrome reported by participants can be understood as part of the ‘doing’ stage, where new nurses are focused on performing tasks and meeting expectations. The need for support and the reliance on peer networks align with the ‘being’ stage, where new nurses seek to establish their professional identity and find their place within the healthcare team.

However, it must be noted that the findings of the study also challenge some aspects of existing models. For example, while Kram's Mentoring Theory (1985) emphasises the importance of mentorship in professional development (Kram 1985), the experiences of ECNs in this study suggest that mentorship alone may not be sufficient. The lack of consistent and effective support from mentors and educators highlights the need for a more comprehensive approach that includes organisational support, adequate staffing, and a positive workplace culture (Moloney et al. 2018; Moloney et al. 2020; Terry et al. 2025). This suggests that existing models may need to be expanded to incorporate these additional factors.

Overall, integrating the findings of the study with established nursing and general transition models enhances our comprehension of the professional transition and workplace adaptation processes experienced by ECNs. These connections not only highlight a more nuanced understanding, but also provide a greater contribution to the field. Nevertheless, future research should be centred on further investigating these theoretical linkages and exploring how existing models can be adapted or expanded to better support ECNs in their transition to professional practice.

## 5 Conclusion

This study sought to explore the experiences of transition from student to nurse among various cohorts of ECNs toward the end of their second year within the nursing profession. A close examination of the experiences of ECNs suggests that there remains work to be undertaken to understand the mechanisms that inform the flow of recently registered nurses out of the workplace or profession altogether. Themes identified ultimately centred around the idea of support. ECNs felt that the differences between anticipated and actual support fostered imposter syndrome and feelings of being overwhelmed. Importantly, as the findings in this study revealed, new nurses are still transitioning into the profession within their second year of practice. Thus, ongoing support past the transitional new graduate 12‐month period is needed. ECNs suggested that their opportunities to link with peers and the empathic nature of the relationship should be capitalised on. While there is further work to be undertaken to explore the deeper dynamics of the relationships between peers and how best to capitalise on this, the possibility of community of practice models may be one key solution. Further, recommendations for policy include the establishment of mandatory mentorship programmes, regular check‐ins with ECNs, ensuring adequate staffing levels, promoting a positive workplace culture, and providing continuous education and leadership training opportunities. Structured mentorship programmes may encompass formal mentorship with structured onboarding, mentorship and cultural training, peer support networks, and systems for regular feedback and programme evaluation. By adopting these strategies, healthcare organisations may enhance the support provided to ECNs, ultimately improving their retention and contributing to a more stable and effective nursing workforce.

## Author Contributions

Liz Ryan: formal analysis; original draft preparation; review and editing. (contribution: 25%); Leah East: formal analysis; original draft preparation; review and editing. (contribution: 25%); Andy Davies: original draft preparation; review and editing. (contribution: 10%); Blake Peck: conceptualisation; investigation; methodology; project administration; visualisation; original draft preparation; review and editing. (contribution: 20%); Daniel Terry: conceptualisation; Data curation; formal analysis; investigation; methodology; project administration; visualisation; original draft preparation; review editing. (contribution: 20%).

## Conflicts of Interest

The authors declare no conflicts of interest.

## Data Availability

The data that support the findings of this study are available on request from the corresponding author. The data are not publicly available due to privacy or ethical restrictions.
